# Enhanced xylose fermentation and hydrolysate inhibitor tolerance of *Scheffersomyces shehatae* for efficient ethanol production from non-detoxified lignocellulosic hydrolysate

**DOI:** 10.1186/s40064-016-2713-4

**Published:** 2016-07-11

**Authors:** Srisuda Senatham, Thada Chamduang, Yotin Kaewchingduang, Anon Thammasittirong, Malee Srisodsuk, Adam Elliston, Ian N. Roberts, Keith W. Waldron, Sutticha Na-Ranong Thammasittirong

**Affiliations:** Department of Microbiology, Faculty of Liberal Arts and Science, Kasetsart University, Kamphaeng Saen Campus, Nakhon Pathom, 73140 Thailand; Microbial Biotechnology Unit, Faculty of Liberal Arts and Science, Kasetsart University, Kamphaeng Saen Campus, Nakhon Pathom, 73140 Thailand; Biorefinery Center, Institute of Food Research, Norwich, NR4 7UA UK; National Collection of Yeast Cultures, Institute of Food Research, Norwich, NR4 7UA UK

**Keywords:** Ethanol, *Scheffersomyces shehatae*, Lignocellulose, Hydrolysate inhibitor, UV-mutagenesis

## Abstract

Effective conversion of xylose into ethanol is important for lignocellulosic ethanol production. In the present study, UV-C mutagenesis was used to improve the efficiency of xylose fermentation. The mutated *Scheffersomyces shehatae* strain TTC79 fermented glucose as efficiently and xylose more efficiently, producing a higher ethanol concentration than the wild-type. A maximum ethanol concentration of 29.04 g/L was produced from 71.31 g/L xylose, which was 58.95 % higher than that of the wild-type. This mutant also displayed significantly improved hydrolysate inhibitors tolerance and increased ethanol production from non-detoxified lignocellulosic hydrolysates. The ethanol yield, productivity and theoretical yield by TTC79 from sugarcane bagasse hydrolysate were 0.46 g/g, 0.20 g/L/h and 90.61 %, respectively, while the corresponding values for the wild-type were 0.20 g/g, 0.04 g/L/h and 39.20 %, respectively. These results demonstrate that *S. shehatae* TTC79 is a useful non-recombinant strain, combining efficient xylose consumption and high inhibitor tolerance, with potential for application in ethanol production from lignocellulose hydrolysates.

## Background

With the increased interests in alternative energy, lignocellulosic biomass is attracting considerable attention as a potential low-cost feedstock for ethanol production. Lignocellulosic biomass is mainly composed of cellulose and hemicellulose. Cellulose is a linear polymer of glucose units linked by β-1-4-glycosidic bonds, whereas hemicellulose is a branched chain of pentoses (xylose and arabinose) and hexoses (glucose, mannose and galactose) (Zaldivar et al. 2001).

Xylose is the second most abundant fermentable sugar in lignocellulosic materials after glucose. Efficient conversion of xylose into ethanol is therefore important for yeast strains used in lignocellulosic ethanol production. *Saccharomyces cerevisiae* is the best-known microorganism used for industrial ethanol fermentation, but this yeast does not naturally ferment pentose sugars to ethanol (Matsushika et al. 2009). Several non-*Saccharomyces* yeasts, such as *Scheffersomyces shehatae* (Syn. *Candida shehatae*), *Scheffersomyces stipitis* (Syn. *Pichia stipitis*) and *Pachysolen tannophilus*, have been found to ferment both glucose and xylose to ethanol and have been investigated for applications in ethanol production (Bajwa et al. 2010; Cheng et al. 2007; Martiniano et al. 2013). *S. shehatae* is one good candidate for sugar mixture fermentation. It is well known that this yeast is Crabtree negative which requires oxygen for growth and produces ethanol under oxygen limited conditions (Hahn-Hägerdal et al. 2006; Tanimura et al. 2015). Nevertheless, a few strains of this yeast, such as *S. shehatae* JCM 18690, have been reported as Crabtree positive (Tanimura et al. 2015). *S. shehatae* showed high performances in terms of yield and productivity using synthetic media (Hickert et al. 2013; Li et al. 2012). However, ethanol production from lignocellulosic residues by *S. shehatae* and other xylose-fermenting yeasts result in a relatively low ethanol yield and productivity. In addition, these yeasts are also sensitive to breakdown compounds in the hydrolysate, such as weak acids, furan derivatives and phenolic compounds which have inhibitory effects on microbial growth and fermentation (Georgieva et al. 2008; Lohmeier-Vogel et al. 1998; Zhang et al. 2011). Consequently, a considerable amount of research has focused on xylose-fermenting yeasts that show high substrate consumption rates and can yield a large amount of ethanol from lignocellulosic biomass such that it would be beneficial to commercial ethanol production. Johannsen et al. (1985) attempted to generate polyploid strains of *S. shehatae* by protoplast fusion. Increasing the level of ploidy from the haploid to the diploid, triploid and tetraploid levels of the fusants resulted in improvement in ethanol production rate from xylose. Li et al. (2012) attempted to improve ethanol production of xylose-fermenting *S. shehatae* ATCC 22984 by UV-mutagenesis. The mutant, Cs3512, showed better fermentation of xylose and mixtures of xylose and glucose. It also showed potential in simultaneous saccharification and fermentation (SSF) of lime-pretreated rice straw achieving 77 % of the theoretical yield. Also using UV-mutagenesis, Hughes et al. (2012) obtained mutant of *S. stipitis* with increased ethanol production and anaerobic growth on lignocellulosic hydrolysate. Pereira et al. (2015) was able to obtain a mutant of *S. stipitis* adapted to hardwood spent sulfite liquor with improved ethanol yield and tolerance to inhibitors. Huang et al. (2009) also obtained an adapted strain of *S. stipitis* with increased ethanol production from rice straw hydrolysate and enhanced inhibitor tolerance.

In this study, we attempted to improve the ethanol production ability from xylose of *S. shehatae* 43CS using UV-mutagenesis followed by selection of mutants having increased ethanol production from xylose using 2,3,5-triphenyltetrazolium chloride (TTC) screening. The selected mutant was characterized and compared with the wild-type, *S. shehatae* 43CS, for its fermentative ability in both synthetic media and in non-detoxified biomass hydrolysate. Additionally, its ability to tolerate inhibitory compounds in lignocellulosic hydrolysate was also investigated.

## Results and discussion

### UV-mutagenesis and selection of improved xylose-fermenting mutants

In order to increase ethanol production from xylose, *S. shehatae* 43CS was subjected to UV-C mutagenesis and selection of mutants by the 2,3,5-triphenyltetrazolium chloride (TTC) method. TTC is a redox indicator that is commonly used for demonstrating activity of dehydrogenases. In the presence of dehydrogenases, the colorless TTC is reduced to a red reductive product formazan (Friedel et al. 1994; Olga et al. 2008). Alcohol dehydrogenases, catalyzing the interconversion of acetaldehyde to ethanol, play an important role in ethanol fermentation. The highly colored formazan of yeast colonies may have relatively high activity of alcohol dehydrogenase which relates to high ethanol fermentation performance. Therefore, the TTC method has been applied to screen high ethanol-producing yeasts (Li et al. 2012). In this study, we selected 90 colonies showing red color on YPX medium covered with TTC agar after incubation for 2 h at 30 °C for primary screening of their ethanol fermentation abilities from xylose. Among these, six mutants were selected based on their higher and faster accumulation of CO_2_ gas in the Durham tubes compared to the wild-type and the other mutants. The result of the shake-flask fermentation was that three of the selected mutants displayed a higher ethanol production than the wild-type. The mutant, designated as TTC79, showed more efficient xylose consumption, ethanol production and concentration compared with the wild-type and the other mutants (Table 1). The maximum ethanol concentration by TTC79 was 17.12 g/L, which was 64.48 % higher than the wild-type strain. The ethanol production ability of TTC79 was not significantly changed even after twenty cycles of growth.

**Table 1:** Ethanol production of mutants and the wild-type in YPX medium containing 50 g/L xylose at 30 °C for 48 h

Strain	Ethanol (g/L)	Residual xylose (g/L)	Ethanol yield (% of theoretical yield)^1^
TTC28	8.80 ± 0.14^c^	9.92 ± 0.38^b^	43.05^c^
TTC79	17.12 ± 0.12^a^	0.16 ± 0.00^d^	67.35^a^
TTC80	12.32 ± 0.12^b^	3.52 ± 0.28^c^	51.97^b^
43CS (wild-type)	6.08 ± 0.16^d^	10.72 ± 0.32^a^	30.35^d^

Different letters indicate significant differences between the yeast strains (*p* < 0.05)

Data represent the mean ± SD from three independent experiments

^1^Theoretical yield of ethanol from xylose is 0.51 g_p_/g_s_, theoretical yield is calculated as ethanol yield multiplied by 100 and divided by 0.51

### Fermentation characterization of *S. shehatae* TTC79 in synthetic medium

**Fig. 1:**
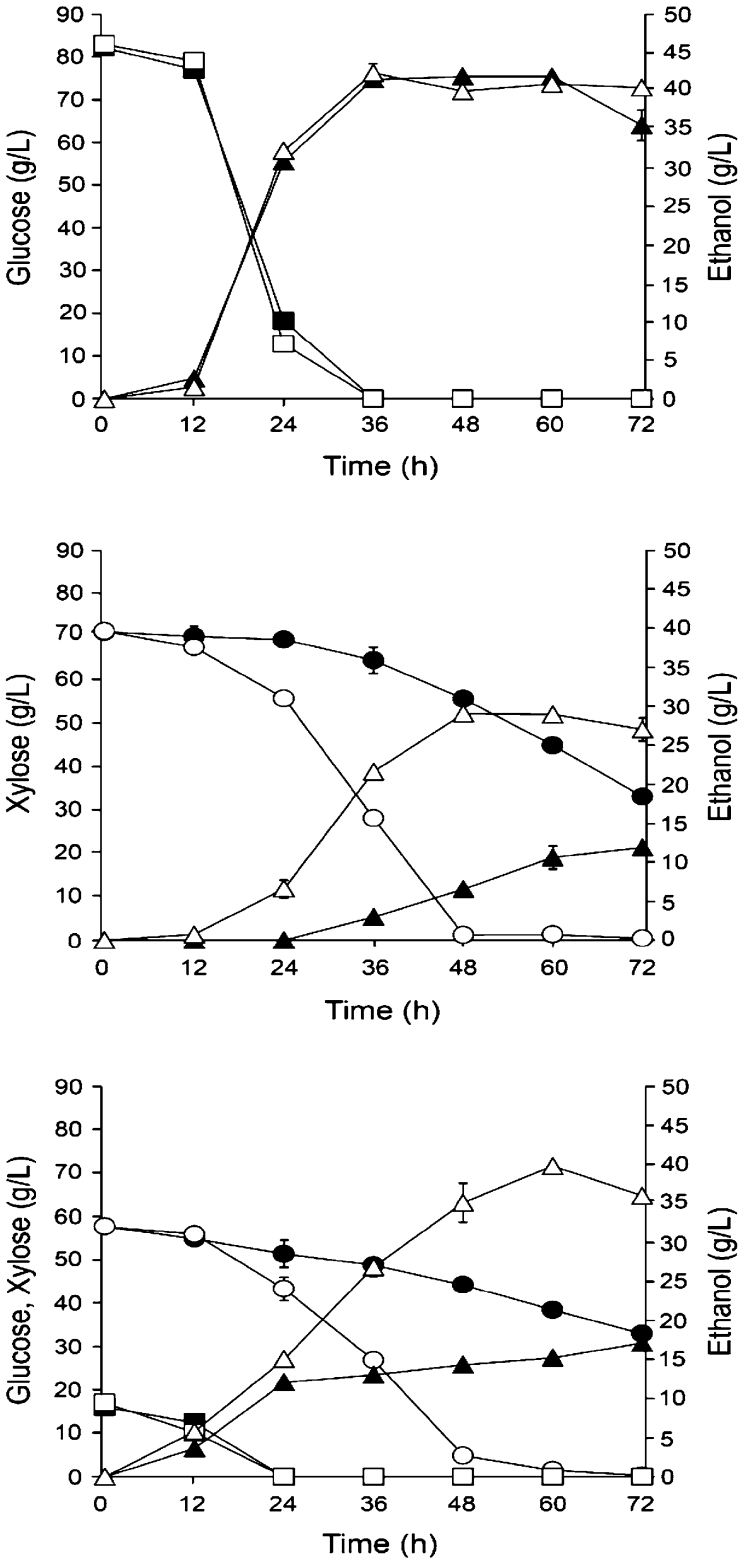
Sugar consumption and ethanol production by TTC79 and the wild-type in synthetic medium containing glucose (**a**) xylose (**b**) and glucose/xylose mixture (**c**). Wild-type/ethanol (*filled triangle*), wild-type/glucose (*filled square*), wild-type/xylose (*filled circle*), TTC79/ethanol (*open triangle*), TTC79/glucose (*open square*), TTC79/xylose (*open circle*). Data represent the mean ± standard deviation from three independent experiments

The ability of TTC79 to ferment glucose, xylose and mixed sugars in synthetic medium was investigated independently by shake-flask studies. The glucose consumption and fermentation patterns for TTC79 were similar to the wild-type (Fig. 1a). Glucose was completely consumed by both TTC79 and the wild-type within 36 h. The maximum range of ethanol concentrations produced by TTC79 and the wild-type was 41.94–42.40 g/L. These results indicated that the glucose fermentation ability was not severely affected by mutations in TTC79.

Xylose was utilized and fermented to ethanol by TTC79 and the wild-type at a slower rate than glucose (Fig. 1b). This pentose sugar was almost completely consumed by TTC79 within 48 h, while the wild-type consumed only 38.06 g/L of the xylose within 72 h. TTC79 produced ethanol from xylose more rapidly and at a higher yield than the wild-type. The maximum ethanol production of 29.04 g/L was obtained for TTC79 at 48 h and that for the wild-type was 11.92 g/L at 72 h. Naturally, xylose-fermenting yeasts, including *S. shehatae*, have been reported to ferment xylose to ethanol and xylitol (Buhner and Agbleror 2004; Li et al. 2012). In this study, xylitol accumulation was observed at very low concentration values by TTC79 (2.35 g/L) and the wild-type (<0.20 g/L) at 72 h (data not shown). With regard to xylose consumption and fermentation of TTC79, these results suggested that higher ethanol production and xylitol production by TTC79 was due to increased efficient xylose consumption.

Under the mixture of glucose and xylose, glucose repression on xylose uptake is a very common among xylose-fermenting yeasts (Bajwa et al. 2010; Lebeau et al. 2007). In this study, glucose underwent fast depletion within the first 24 h by TTC79 and the wild-type. Xylose consumption occurred simultaneously to glucose consumption, and then xylose was rapidly consumed after glucose depletion. This pentose sugar was almost completely consumed by TTC79 within 60 h, while the wild-type consumed only 24.78 g/L of the xylose within 72 h. The maximum ethanol production of 39.84 g/L was obtained at 60 h by TTC79, whereas 17.12 g/L ethanol was obtained by the wild-type at 72 h. The xylitol production during fermentation was very low, only 0.85 g/L was observed by TTC79 at 72 h (data not shown). The results in this study clearly demonstrated that TTC79 increased efficient xylose consumption while maintaining high glucose consumption ability, leading to improved ethanol production from the glucose-xylose mixture.

### Growth tolerance of *S. shehatae* TTC79 in the presence of acetic acid, furfural and 5-hydroxymethy furfural (HMF)

**Fig. 2:**
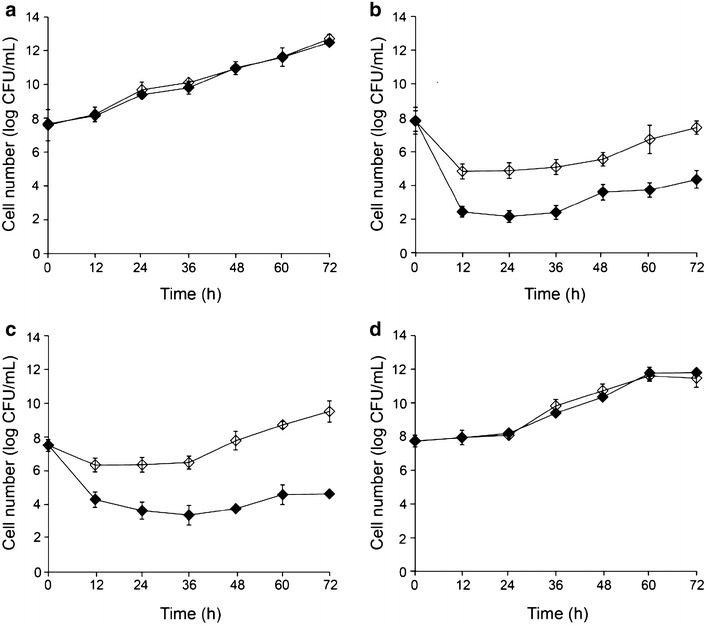
Cell viability of TTC79 and the wild-type in the absence (**a**) and in the presence of 5.25 g/L acetic acid (**b**), 1.75 g/L furfural (**c**) and 1.30 g/L HMF (**d**). Wild-type (*filled diamond*), TTC79 (*open diamond*). Data represent the mean ± SD from three independent experiments

Acetic acid, furfural and HMF are among the most potent inhibitors found in lignocellulosic hydrolysates (Klinke et al. 2004; Taherzadeh et al. 2000). These compounds are known to inhibit microbial growth, sugar consumption and therefore affect ethanol fermentation performance (Georgieva et al. 2008; Zhang et al. 2011). To determine if TTC79 would also exhibit enhanced tolerance to acetic acid, furfural and HMF, its growth tolerance was performed and compared with that of the wild-type by measuring cell viability in the presence of individual inhibitors. The concentrations of these inhibitors used in this study were similar to or higher than those reported in lignocellulosic hydrolysates (Agbogbo and Wenger 2007; Bajwa et al. 2009; Larsson et al. 1999). In the absence of inhibitor, no difference of growth pattern was observed between TTC79 and the wild-type (Fig. 2a). In the presence of 5.25 g/L acetic acid, cell number of TTC79 and the wild-type declined in the first 12 h by about 3 log units and 5 log units, respectively, and then TTC79 grew at a faster rate as compared to the wild-type (Fig. 2b). At 1.75 g/L furfural, TTC79 was capable of growing after lag phase of 36 h (Fig. 2c). In contrast, the wild-type was able to remain viable in 1.75 g/L furfural, but no increase in cell number was observed. The results of cell viability in the presence of 1.30 g/L HMF revealed that no difference was seen between TTC79 and the wild-type in their growth responses (Fig. 2d).

Generally, yeast cell growth was inhibited at an acetic acid concentration of around 2.00–5.00 g/L (Bajwa et al. 2009, 2010; Larsson et al. 1999). Furfural and HMF are the inhibitors produced from pentose and hexose sugars degraded during acid hydrolysis. It was found that 0.90–2.00 g/L furfural in hydrolysate was able to reduce fermentation rate and/or stop yeast growth (Agbogbo and Wenger 2007; Bajwa et al. 2010; Huang et al. 2009). HMF concentrations of 0.50 g/L or higher have been reported to inhibit yeast growth (Agbogbo and Wenger 2006; Bajwa et al. 2010). It has been reported that pentose-fermenting yeasts including *S. shehatae* are susceptible to the inhibitors generated during the diluted acid pretreatment of plant biomass (Huang et al. 2009; Lohmeier-Vogel et al. 1998). According to the cell viability of *S. shehatae* TTC79 in the presence of individual inhibitors, it was evident that TTC79 exhibited enhanced tolerance to the inhibitors in lignocellulosic hydrolysate compared to the wild-type. Efficient xylose fermentation and tolerance of toxic compounds are polygenic traits arising via complex mechanisms (Demeke et al. 2013; Wang et al. 2014; Zhao and Bai 2009). Improved understanding of the intracellular responses and mechanisms of TTC79 to inhibitory compounds and the synergistic effect of these inhibitors on yeast cell metabolism during lignocellulosic ethanol production will enable superior strains for efficient lignocellulosic ethanol production to be developed.

### Fermentation characterization of *S. shehatae* TTC79 in non-detoxified hydrolysate

**Fig. 3:**
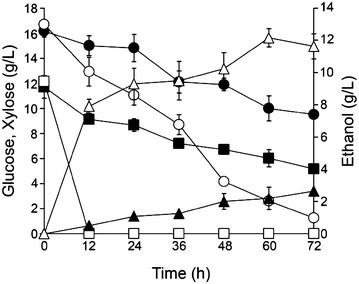
Sugar consumption and ethanol production by TTC79 and the wild-type in non-detoxified sugarcane bagasse hydrolysate. Wild-type/ethanol (*filled triangle*), wild-type/glucose (*filled square*), wild-type/xylose (*filled circle*), TTC79/ethanol (*open triangle*), TTC79/glucose (*open square*), TTC79/xylose (*open circle*). Data represent the mean ± SD from three independent experiments

In addition to pentose and hexose sugars, the numerous types of inhibitors are produced during acid hydrolysis process and usually a detoxification step is needed to improve fermentability. Detoxification results in sugar loss and increase production cost (Buhner and Agblevor 2004). Xylose-fermenting yeast with high inhibitor tolerance that is able to ferment non-detoxified hydrolysate to ethanol would be very attractive for commercial lignocellulosic ethanol production. The results in Fig. 3 showed that simultaneous consumption of glucose with xylose was observed in TTC79 and the wild-type. TTC79 consumed the sugar mixture in undetoxified sugarcane bagasse hydrolysate containing 6.45 g/L acetic acid, 0.28 g/L furfural and 1.60 g/L HMF to a greater extent than the wild-type and this led to higher ethanol production. The maximum ethanol concentration, yield and the theoretical yield by TTC79 were 12.15 g/L, 0.46 g/g and 90.61 %, respectively (Table 2). Ethanol productivity of TTC79 was also considerably faster than the wild-type. The maximum ethanol productivity by TTC79 was 0.20 g/L/h, while the wild-type showed productivity of 0.04 g/L/h. No xylitol production was detected in this fermentation (data not shown). One possible explanation might be that the delay in consumption rate of xylose in the hydrolysate, xylitol therefore could be completely converted to ethanol.

**Table 2:** Ethanol production by TTC79 and the wild-type from non-detoxified sugarcane bagasse hydrolysates^1^ at 30 °C

	TTC79	Wild-type
Maximum ethanol concentration (g/L)	12.15 ± 1.57^a^	2.64 ± 0.09^b^
Ethanol yield^2^ (g_p_/g_s_)	0.46 ± 0.06^a^	0.20 ± 0.06^b^
Theoretical yield^3^ (%)	90.61 ± 0.58^a^	39.20 ± 0.51^b^
Fermentation time^4^ (h)	60	72
Ethanol productivity (g/L/h)	0.20 ± 1.55^a^	0.04 ± 0.01^b^

Different letters indicate significant differences between yeast strains (*p* < 0.05)

Data represent the mean ± SD from three independent experiments

^1^Fermentable sugars in hydrolysate: glucose: 12.15 g/L, xylose: 16.70 g/L

^2^Ethanol yield (g_p_/g_s_) is the calculated as ethanol accumulation divided by glucose and xylose consumed

^3^Theoretical yield of ethanol from glucose is 0.51 g_p_/g_s_ and xylose is 0.51 g_p_/g_s_, theoretical yield is calculated as ethanol yield multiplied by 100 and divided by 0.51

^4^The time points indicate the maximum ethanol concentrations produced by the yeast strains

Generally, several wild-type and mutant of xylose-fermenting yeast strains have been reported to ferment xylose with satisfactory yield in detoxified hydrolysates. Martiniano et al. (2013) found ethanol yield, 0.30 g/g, and ethanol productivity, 0.15 g/L/h, from *S. shehatae* CGS8BY using sugarcane bagasse hydrolysate detoxification by activated charcoal. Cheng et al. (2007) reported that the ethanol yield and ethanol productivity of 0.35 g/g and 0.59 g/L/h using the detoxified sugarcane bagasse hydrolysate by *P. tannophilus* DW06 DSM3651. Huang et al. (2009) reported the ethanol yield using *S. stipitis* BCRC21777 and the adapted *S. stipitis* with detoxified rice straw achieved 0.40 g/g and 0.44 g/g, respectively. However, some studies have reported on the efficient ability of yeast strains to produce ethanol from non-detoxified hydrolysate. Agbogbo et al. (2008) reported the ethanol yields, 0.38–0.42 g/g, from *S. stipitis* CBS6054 using corn stalk without detoxification. Huang et al. (2009) obtained the adapted strain of *S. stipitis* with high ethanol yield, 0.44 g/g, by fermenting non-detoxified rice straw hydrolysate. Wan et al. (2012) obtained ethanol yield of 0.43 g/g, corresponding to 85.10 % of the theoretical yield from cocultures of *S. cerevisiae* Y5 and *S. stipitis* CBS6054. Although, it is difficult to directly compare the results of ethanol production between different studies, it is still useful to display the competitiveness of this yeast strain, *S. shehatae* TTC79, for lignocellulosic ethanol production.

## Conclusion

Most lignocellulosic biomass feedstock contains a significant amount of xylan that is converted to xylose by hydrolysis. High consumption and fermentation of pentose sugars present in lignocellulosic biomass is an important factor to make ethanol production commercially feasible. In the present study, the increased xylose fermentation yeast strain was successfully obtained through UV-C mutagenesis. The *S. shehatae* TTC79 mutant exhibited excellent xylose consumption and fermentation both with xylose alone and with sugars mixture. This mutant also showed high resistance to lignocellulosic inhibitors along with high ethanol yield from dilute-acid lignocellulosic hydrolysate without the need for detoxification. These results demonstrate that *S. shehatae* TTC79 is one of the most efficient non-recombinant strains for lignocellulosic ethanol production described to date.

## Methods

### Yeast strain

The stock culture of *S. shehatae* 43CS from our laboratory culture collection was maintained on YPX agar (10 g/L yeast extract, 20 g/L peptone, 2 g/L xylose) at 4 °C.

### UV-C mutagenesis and mutant selection

UV-mutagenesis was carried out according to Thammasittirong et al. (2013) except that yeast cell suspension was spread on YPX medium. Following UV-treatment, the grown colonies were covered with 2,3,5-triphenyltetrazolium chloride (TTC) (Sigma-Aldrich, St. Louis, USA) agar containing 0.5 g/L xylose, 10 g/L agar and 0.05 g/L TTC (Li et al. 2012). After solidification, the TTC agar-covered plates were incubated at 30 °C for 2 h. The red colonies were selected for xylose fermentation evaluation. The mutant selection experiment was performed in two steps. First, a loopfull of 24 h YPX-grown culture of each mutant was inoculated in 5 mL YPX medium in a test tube containing a Durham tube and incubated at 30 °C for 10 days. Those strains showing high accumulation of CO_2_ gas in the Durham tubes were selected for screening of mutant strains with high ethanol production ability. YPX medium containing 50 g/L xylose was inoculated with overnight YPX cultures to achieve a cell density of 5 × 10^5^ cells/mL. The cultures in Erlenmeyer flasks plugged with cotton were incubated at 30 °C in a shaking incubator under oxygen limited condition, 100 rpm, for 48 h. The mutant that showed the best xylose fermentation ability was selected for further studies.

### Fermentation of sugars in synthetic medium

The selected mutant and wild-type were investigated for their abilities to utilize and ferment glucose (80 g/L) and xylose (80 g/L) individually and 20 g/L glucose/60 g/L xylose mixture. The 24 h pre-cultivated yeast cells in YPX medium were inoculated into 100 mL synthetic medium containing 10 g/L yeast extract, 20 g/L peptone and sugar concentrations as described above in 250 mL Erlenmeyer flasks plugged with cotton. The initial cell concentration was adjusted to cell density of 5 × 10^5^ cells/mL. Fermentations were performed for 72 h at 30 °C in a shaking incubator under oxygen limited condition, 100 rpm. Fermentation samples were withdrawn every 12 h for measurement of cell concentrations, sugar and ethanol analysis. All experiments were performed in three independent experiments.

### Determination of inhibitors tolerance

Yeasts were inoculated in 65 mL YPX medium containing 5.25 g/L acetic acid, 1.75 g/L furfural and 1.30 g/L HMF individually to achieve an initial cell density of 1 × 10^7^ cells/mL. The cultures were incubated at 30 °C with shaking at 100 rpm for 72 h. The appropriate dilutions of each culture were taken for measurement of viable cells using a NucleoCounter YC-100 automated cell counter unit (Chemometec, Inc., Allerød, Denmark).

### Preparation of sugarcane bagasse hydrolysate by dilute-acid hydrolysis

The sun-dried chopped sugarcane bagasse was milled to a particle size 3–5 mm and dried at 60 °C for 24 h. The oven-dried milled bagasse was soaked in 1 % H_2_SO_4_, in a solid–liquid proportion of 1:10, at ambient temperature for 30 min. Acid hydrolysis was performed at 121 °C for 30 min. The hydrolysate was separated from the bagasse solid fraction by filtration. The hydrolysate was neutralized with CaO to pH 5.5 and then centrifuged at 5000×*g* for 5 min to remove the solid. The precipitate formed was removed by vacuum filtration. The hydrolysate was supplemented with 5 g/L KH_2_PO_4_, 2 g/L (NH_4_)_2_SO_4_, 0.2 g/L MgSO_4_·7H_2_O, 1 g/L peptone, 5 g/L yeast extract and finally the pH of hydrolysate was adjusted to 5.5 and autoclaved at 110 °C for 15 min. Sugars and hydrolysate inhibitors in the hydrolysate were analyzed by high-performance liquid chromatography (HPLC).

### Fermentation of the non-detoxified sugarcane bagasse hydrolysate

The 24 h pre-cultivated yeast cells in YPX medium were inoculated into hydrolysate medium with cells initially adjusted to cell density of 1 × 10^7^ cells/mL. Fermentations were carried out at 30 °C as described above. Fermentation samples were taken every 12 h for determining ethanol concentration and sugar concentration in the culture. All experiments were performed in three independent experiments.

### Analytical methods

The ethanol and sugar concentrations were analyzed by Waters 600E HPLC system (Waters Inc., Milford, USA) using a sugar pak I column at 85 °C and a refractive index detector. The mobile phase was deionized water at a flow rate of 0.5 mL/min. Furfural (Sigma-Aldrich, St. Louis, USA), HMF (Sigma-Aldrich, St. Louis, USA) and acetic acid (Merck, Darmstadt, Germany) were separated on C18 column at 25 °C and UV detector. Furfural and HMF were eluted with 20 % acetonitrile in deionized water (80 %) at a flow rate of 1.1 mL/min. Acetic acid was eluted with 1 % acetonitrile in 0.05 M KH_2_PO_4_ (99 %) at a flow rate of 1.0 mL/min.
